# Intrapreneurship Among Nurses in Private Hospitals According to Demographic and Professional Characteristics: A Cross-Sectional Study in Türkiye

**DOI:** 10.3390/healthcare14213684

**Published:** 2026-10-30

**Authors:** Berna Eren, Güllüzar Bozkır, Saliha Koç Aslan

**Affiliations:** 1 Department of Healthcare Management, Faculty of Health Sciences, Acibadem Mehmet Ali Aydinlar University, 34752 İstanbul, Türkiye; 2 Department of Healthcare Management, Graduate School of Health Sciences, Acibadem Mehmet Ali Aydinlar University, 34752 İstanbul, Türkiye; 3 Nursing Services Directorate, Acibadem Healthcare Group, 34718 İstanbul, Türkiye; gulluzar.bozkir@acibadem.com; 4 Medical Services, Acibadem Healthcare Group, 34758 İstanbul, Türkiye; skoc@acibadem.com

**Keywords:** nurse intrapreneurship, nursing, innovation, autonomy, private hospitals, Türkiye, cross-sectional study

## Abstract

**Background/Objectives:** Nursing intrapreneurship is increasingly recognized as a pathway for innovation, quality improvement, and organizational development in healthcare. This study examined nurses’ intrapreneurship levels according to selected demographic and professional characteristics in private hospitals in Türkiye. **Methods:** This descriptive cross-sectional study used an online survey distributed to nurses working in public and private hospitals between December 2023 and April 2024. Because public-sector participation was insufficient for meaningful analysis, the final analytic sample was restricted after data collection to 640 nurses employed in private hospitals. Data were collected using a demographic and professional information form and the 21-item Intrapreneurship Scale. The total scale score was treated as the primary outcome, while subscale analyses were secondary and exploratory. Descriptive statistics, non-parametric tests, Spearman’s rho correlation analysis, and multivariable general linear models were used. **Results:** The mean total Intrapreneurship Scale score was 3.69 ± 0.61. In secondary analyses, innovativeness had the highest mean score (4.27 ± 0.67), followed by risk-taking and proactiveness (3.80 ± 0.69), while autonomy had the lowest score (3.04 ± 0.85). In multivariable models, gender remained associated with the total score and, in secondary analyses, with risk-taking and proactiveness and autonomy; however, adjusted differences were small. No other fixed factor showed a statistically significant overall association after adjustment. **Conclusions:** Nurses working in private hospitals in Türkiye had total intrapreneurship scores above the scale midpoint. Findings should be interpreted in light of the private-hospital restriction, the exploratory nature of subscale analyses, and the role of organizational context in supporting nurse intrapreneurship.

## 1. Introduction

Health systems around the world are under increasing pressure due to population ageing, the growing burden of chronic diseases, rapid technological change, workforce challenges, rising patient expectations for personalized and high-quality care, and escalating healthcare costs. These pressures require healthcare organizations to develop efficient, cost-effective, and sustainable solutions that improve the quality, affordability, accessibility, efficiency and equity of health services [1,2].

In this context, entrepreneurship has become increasingly relevant to transformation in the health sector, as it supports innovative and business-oriented strategies to address complex healthcare challenges, and contributes to the development of new ideas, services, medical technologies, devices and therapies, models of healthcare delivery, and management innovations that can improve healthcare efficiency and support the delivery of affordable care [2,3,4]. In recent decades, health professionals have become ever more engaged in entrepreneurial activities, striving to improve both their organizations and the communities they serve. Their entrepreneurial efforts offer promising solutions to the pressing challenges confronting the health sector, while questioning established practices and encouraging cultural transformation [5,6,7,8,9,10].

Although entrepreneurship has traditionally been associated with the creation of new ventures, its principles are also applicable within established organizations through intrapreneurship. In healthcare organizations, intrapreneurship is particularly important because innovation often depends not only on external entrepreneurial activity but also on the ability of professionals within the system to identify problems or opportunities, develop innovative solutions, and improve processes, services, and care practices from within [11,12,13]. Organizational conditions such as autonomy, managerial support, participation in decision-making, and recognition of innovative efforts can strengthen employees’ intrapreneurial behavior and contribute to long-term strategic growth [14,15,16].

Nurses are key contributors to healthcare delivery and organizational quality improvement. They play a vital role in ensuring patient safety and consistent delivery of high-quality, accessible, evidence-based, and patient-centered care. Beyond direct patient care, nurses participate in organizational quality and safety initiatives, support shared decision-making practices that are responsive to patients’ diverse needs, and demonstrate clinical leadership within multidisciplinary teams [17,18,19]. As nurses’ roles and responsibilities evolve in response to demographic, technological, clinical, and policy-related changes, nursing entrepreneurship has gained attention as a pathway for nurses to participate in innovation and change-oriented practices [5,7,8,19,20,21]. The International Council of Nurses (ICN) [22] defines a nurse entrepreneur as “a proprietor of a business that offers nursing services of a direct care, educational, research, administrative, or consultative nature”. Entrepreneurial activities foster nurses’ personal growth, autonomy, confidence, flexibility, innovation, and proactivity, while also contributing to the strengthening and advancement of the profession itself [7,20,21,23,24].

Nurses may engage in entrepreneurship or intrapreneurship across private, public, or hybrid health systems. Although these concepts share common foundations, they differ in terms of context, scope, and organizational boundaries. Nurse entrepreneurs typically operate outside traditional employment structures by establishing independent practices, consultancy or educational services, or other health-related ventures, whereas nurse intrapreneurs work within existing healthcare organizations and apply entrepreneurial skills to identify care-related problems, propose new solutions, improve service processes, take initiative, and contribute to organizational innovation. In this regard, examining intrapreneurship among nurses shifts the focus from external business creation to the internal capacity of healthcare organizations to support nurses’ innovation, autonomy, proactiveness, and calculated risk-taking. Although nurse intrapreneurs may face lower uncertainty and personal financial risk than independent nurse entrepreneurs because they operate within institutional structures, they may still encounter barriers such as limited autonomy, hierarchical decision-making, resource constraints, and organizational resistance to change [11,19,25,26,27,28,29].

Despite the growing recognition of entrepreneurship and intrapreneurship in nursing, empirical evidence and comparative statistical data remain limited and inconsistent. Available global estimates suggest that only 0.5–1% of working nurses are engaged in entrepreneurship, with considerable geographical variation [7,28,30]. By contrast, comparable population-level estimates for nurse intrapreneurs appear to be unavailable, highlighting a significant gap in the literature.

Previous studies have more often focused on entrepreneurship or entrepreneurial intention than on intrapreneurship within organizational settings, particularly in relation to nurses’ entrepreneurial tendencies, entrepreneurship education, individual innovation, and entrepreneurship-related characteristics [21,23,31,32]. Several studies suggest that entrepreneurial tendencies may be associated with individual and professional characteristics [23,31,32,33]. However, the reported associations are not uniform across studies. For example, Soysal and Kolca found no significant differences in nurses’ intrapreneurship according to educational level, age, gender, or marital status [34], whereas Erbir and Yılmaz reported higher intrapreneurship among younger and single healthcare workers but no significant gender difference [35]. These contrasting findings suggest that demographic associations may vary according to the study population and context. Evidence is even more limited regarding employed nurses working in healthcare organizations, where intrapreneurship may also be related to institutional roles, work environments, and opportunities for innovation.

Demographic and professional characteristics such as age, gender, education, work experience, position, and work unit may affect nurses’ opportunities, autonomy, confidence, and willingness to engage in intrapreneurial behavior [23,32,33,34]. Understanding these associations is important for identifying professional groups that may require greater organizational support and for developing strategies to strengthen nurses’ innovation capacity across different workforce profiles. Therefore, this study aimed to examine whether nurses’ intrapreneurship levels differed according to selected demographic and professional characteristics among nurses employed in private hospitals in Türkiye. By focusing specifically on intrapreneurship rather than general entrepreneurship, this study contributes to understanding how nurses’ innovation-oriented characteristics are expressed within the organizational context of private hospitals.

## 2. Materials and Methods

This descriptive and cross-sectional study aimed to examine nurses’ intrapreneurship levels according to their demographic and professional characteristics. The study was conducted between December 2023 and April 2024 using an online survey distributed to nurses working in public and private hospitals in Türkiye. A convenience sampling method was used, and no a priori power analysis was conducted to estimate the sample size. Participation was based on nurses’ availability and willingness to take part in the study. The inclusion criterion was being actively employed as a nurse during the data collection period. Nurses who voluntarily agreed to participate by confirming the informed consent statement at the beginning of the online questionnaire were included in the study and no further sample selection procedure was applied.

Data were collected using a structured questionnaire consisting of two sections. The first section included questions on participants’ demographic and professional characteristics. The second section included the Intrapreneurship Scale, originally developed by Naktiyok [36] and revised and validated by Durmaz [37]. Although the scale was not originally developed specifically for nurses or healthcare professionals, it has been used in previous research involving healthcare workers [35], and its internal consistency was evaluated in the present sample using Cronbach’s alpha coefficients. The scale consists of 21 items and three subscales: innovativeness, risk-taking and proactiveness, and autonomy. Items are rated on a five-point Likert-type scale ranging from 1 = strongly disagree to 5 = strongly agree. Higher scores indicate higher levels of intrapreneurship. In this study, subscale scores were calculated by averaging the items within each subscale, and the total scale score was calculated by averaging all scale items. The study variables and questionnaire items are provided in Appendix A.

Ethical approval for the study was obtained from the Acibadem University and Acibadem Healthcare Institutions Medical Research Ethics Committee (ATADEK) [2023-19/662; 30 November 2023]. Permission to use the scale was obtained from Durmaz [37], and informed consent was obtained electronically from all participants before data collection. The questionnaire was distributed electronically through professional nursing communication groups and e-mail lists, and responses were collected online. These channels were used solely for recruitment, and the survey did not record the specific channel through which each respondent accessed the questionnaire. Recruitment was not restricted to a single healthcare group. Although the initial data collection plan included nurses from both public and private hospitals, public-sector participation was insufficient for meaningful analysis; therefore, the present study was restricted to nurses working in private hospitals. Because the exact number of nurses reached through these channels could not be determined, the response rate could not be calculated. To protect participant anonymity and reduce concerns about institutional identification, the questionnaire did not collect respondents’ province of employment, hospital names, or healthcare-group identifiers; therefore, the geographic and institutional distribution of the final sample could not be determined. All submitted questionnaires were complete because the online survey required responses to all items before submission.

Data were analyzed using the Statistical Package for the Social Sciences (SPSS) version 23.0. Categorical variables were presented as frequencies and percentages, while continuous variables were presented as mean, standard deviation, median, interquartile range, minimum, and maximum values. To further examine the construct validity of the Intrapreneurship Scale in the present nurse sample, exploratory factor analysis was performed using principal axis factoring. Sampling adequacy was evaluated using the Kaiser–Meyer–Olkin measure, and the suitability of the correlation matrix was assessed using Bartlett’s test of sphericity. Because the original scale includes three dimensions, a three-factor solution was examined, and factor interpretability was evaluated based on the rotated pattern matrix. The internal consistency of the scale and its subscales was evaluated using Cronbach’s alpha coefficient.

The normality of the total scale and subscale scores was assessed using the Kolmogorov–Smirnov and Shapiro–Wilk tests, skewness and kurtosis values, and visual inspection of histograms and Q–Q plots. Since the data did not meet the normality assumption, the Mann–Whitney U test was used to compare scale and subscale scores between two groups, and the Kruskal–Wallis test was used for comparisons among three or more groups. Since the Kruskal–Wallis test indicates whether a statistically significant difference exists among three or more groups but does not identify which specific groups differ, pairwise Mann–Whitney U tests were performed as post-hoc comparisons when the Kruskal–Wallis test was significant. Bonferroni correction was applied to control the family-wise Type I error rate arising from multiple pairwise comparisons. Spearman’s rho correlation analysis was used to examine the relationships between the total Intrapreneurship Scale score and its subscale scores. To address the potential interrelationships among demographic and professional characteristics, multivariable general linear models were additionally performed. Separate models were constructed for the total Intrapreneurship Scale score and each subscale score as dependent variables. Given the limited replication of the original three-factor structure in the exploratory factor analysis, the total Intrapreneurship Scale score was designated as the primary outcome, whereas analyses of the three subscales were treated as secondary, exploratory analyses. Gender, age group, educational level, marital status, unit of employment, professional position, and professional experience were entered simultaneously as fixed factors. This approach was used to examine whether the associations observed in the bivariate analyses remained after adjustment for relevant demographic and professional characteristics. Type III sums of squares were used, and partial eta squared values were reported as measures of effect size. Model diagnostics were also examined for the multivariable general linear models. Predicted values, unstandardized residuals, standardized residuals, and residual plots were evaluated, and the homogeneity of error variances was assessed using Levene’s test. Because some models showed evidence of unequal error variances, the adjusted findings were interpreted cautiously, with emphasis on effect sizes, confidence intervals, and the direction of associations rather than statistical significance alone. A *p* value of less than 0.05 was considered statistically significant for the main analyses. For post-hoc pairwise comparisons, Bonferroni-adjusted significance thresholds were used; therefore, *p* ≤ 0.008 was considered statistically significant for variables with four groups, and *p* ≤ 0.005 was considered statistically significant for variables with five groups.

The reporting of this study was guided by the STROBE checklist for cross-sectional studies.

## 3. Results

A total of 640 nurses were included in the analysis. The majority of participants were female (83.0%). The mean age was 32.1 years (SD = 7.9), with a median age of 29.0 years (range: 21–60), and the largest age group was 26–30 years (39.2%). The proportions of single and married nurses were similar (49.7% and 50.3%, respectively). Regarding educational level, participants were fairly evenly distributed across vocational health high school nursing graduates (31.6%), bachelor’s degree holders (31.4%), and associate degree holders (25.5%), while 11.6% had a postgraduate degree. Nurses were employed in diverse clinical settings, with the largest proportion working in inpatient units (34.4%), followed by outpatient units (24.4%), intensive care (17.3%), emergency departments (12.5%), and management units (11.4%). Nearly half of the participants were staff nurses (48.9%), and 7.3% were nurse managers, with the remaining participants working as team leaders or charge nurses. Work experience varied considerably, with nearly half of participants (46.7%) having 121 months or more of professional experience. The demographic and professional characteristics of the nurses are presented in Table 1.

Descriptive statistics for the Intrapreneurship Scale and its subscales are shown in Table 2. The mean total Intrapreneurship Scale score was 3.69 ± 0.61. Among the subscales, the highest mean score was observed for innovativeness (4.27 ± 0.67), followed by risk-taking and proactiveness (3.80 ± 0.69), while the lowest mean score was observed for autonomy (3.04 ± 0.85).

Cronbach’s alpha coefficients were 0.915 for the total Intrapreneurship Scale, 0.892 for innovativeness, 0.893 for risk-taking and proactiveness, and 0.811 for autonomy, indicating good to excellent internal consistency.

Exploratory factor analysis was conducted to further examine the construct validity of the Intrapreneurship Scale in this nurse sample. The Kaiser–Meyer–Olkin value was 0.929, and Bartlett’s test of sphericity was statistically significant (χ^2^ = 7388.364, df = 210, *p* < 0.001), indicating that the data were suitable for factor analysis. Three factors had eigenvalues greater than 1.00, and the three-factor solution explained 52.8% of the total variance. However, the rotated pattern matrix did not clearly reproduce the original three-dimensional structure of the scale in this sample. Most items loaded predominantly on the first factor, while the second factor mainly included autonomy-related items, and the third factor did not form a clearly interpretable independent dimension. Therefore, the original subscale scoring was retained for comparability with previous studies, but the factor-analytic findings were interpreted as indicating limited replication of the original factor structure in the present sample. Accordingly, the total Intrapreneurship Scale score was treated as the primary outcome in subsequent analyses, whereas findings for the three subscales were considered secondary and exploratory. The rotated factor-loading matrix, eigenvalues, and communalities are provided in Supplementary Table S1.

The comparison of the Intrapreneurship Scale and subscale scores according to demographic and professional characteristics, including median and interquartile range values, is presented in Table 3. Gender was significantly associated with total intrapreneurship, risk-taking and proactiveness, and autonomy scores, while marital status was significantly associated with total intrapreneurship and autonomy scores. Autonomy differed significantly according to age group, position, and professional experience; innovativeness differed significantly according to educational level; and risk-taking and proactiveness differed significantly according to position. No significant differences were found in any scale or subscale according to unit of employment.

Pairwise Mann–Whitney U tests with Bonferroni correction were performed for variables with significant Kruskal–Wallis test results. Post hoc analyses showed that nurses aged ≤25 years had significantly higher autonomy scores than those aged 31–39 years (*U* = 6632.500, *Z* = 2.796, *p* = 0.005) and those aged ≥40 years (*U* = 5705.500, *Z* = −4.027, *p* < 0.001). Nurses aged 26–30 years also had significantly higher autonomy scores than those aged ≥40 years (*U* = 13,310.000, *Z* = −3.063, *p* = 0.002). Regarding educational level, nurses with a postgraduate degree had significantly higher innovativeness scores than vocational health high school nursing graduates (*U* = 5678.500, *Z* = −3.090, *p* = 0.002). In terms of position, staff nurses had significantly higher total intrapreneurship scores (*U* = 21,316.000, *Z* = −2.649, *p* = 0.008) and autonomy scores (*U* = 19,957.500, *Z* = −3.621, *p* < 0.001) than charge nurses. For professional experience, nurses with 13–36 months of experience had significantly higher autonomy scores than those with ≥121 months of experience (*U* = 6990.500, *Z* = −4.032, *p* < 0.001). Although risk-taking and proactiveness scores differed significantly according to position in the overall Kruskal–Wallis test, no pairwise comparison remained significant after Bonferroni correction.

Overall, the bivariate analyses showed that nurses’ intrapreneurship levels differed according to selected demographic and professional characteristics. The most consistent unadjusted differences were observed in the autonomy subscale, which varied by gender, marital status, age group, position, and professional experience. However, given the potential interrelationships among these characteristics, additional multivariable analyses were conducted.

The results of the multivariable general linear models are presented in Table 4. Model diagnostics indicated that Levene’s test was significant for the total Intrapreneurship Scale, innovativeness, and risk-taking and proactiveness models, but not for autonomy; therefore, the adjusted findings were interpreted cautiously, with particular attention to effect sizes, 95% confidence intervals, and the direction of associations. After adjustment for gender, age group, educational level, marital status, unit of employment, professional position, and professional experience, gender remained significantly associated with the total intrapreneurship score (F = 7.697, *p* = 0.006, partial η^2^ = 0.012), risk-taking and proactiveness (F = 8.759, *p* = 0.003, partial η^2^ = 0.014), and autonomy (F = 14.235, *p* < 0.001, partial η^2^ = 0.022). However, these effect sizes were small. In the parameter estimates, female nurses scored lower than male nurses on the total scale (B = −0.186, 95% CI: −0.317 to −0.054), risk-taking and proactiveness (B = −0.226, 95% CI: −0.375 to −0.076), and autonomy (B = −0.344, 95% CI: −0.523 to −0.165). No demographic or professional characteristic remained significantly associated with innovativeness in the adjusted model. At the overall factor level, no demographic or professional characteristic other than gender remained significantly associated with the study outcomes after adjustment. Complete results for all fixed factors, including F statistics, *p* values, partial η^2^, category-specific parameter estimates with 95% CIs, and overall model R^2^ values, are provided in Supplementary Table S2.

Correlations between the Intrapreneurship Scale and its subscales are presented in Table 5. Spearman’s rho correlation analysis showed positive and statistically significant correlations between the total scale score and all subscale scores. As expected, the strongest correlation was observed between the total scale score and risk-taking and proactiveness (r = 0.906, *p* < 0.001), followed by autonomy (r = 0.749, *p* < 0.001) and innovativeness (r = 0.585, *p* < 0.001). Correlations among the subscales ranged from weak to moderate (r = 0.139–0.564, *p* < 0.001).

## 4. Discussion

### 4.1. Main Findings and Interpretation

This study examined nurses’ intrapreneurship levels according to their demographic and professional characteristics among nurses employed in private hospitals in Türkiye. The primary outcome, the total Intrapreneurship Scale score, was above the scale midpoint. In secondary, exploratory analyses, innovativeness had the highest mean score and autonomy had the lowest mean score. In the bivariate analyses, selected intrapreneurship scores differed according to gender, marital status, age group, educational level, position, and professional experience, whereas no significant differences were observed according to unit of employment. However, at the overall factor level, gender remained the only characteristic significantly associated with the total intrapreneurship score and, in secondary exploratory analyses, with risk-taking and proactiveness and autonomy. These findings suggest that some differences observed in the bivariate analyses may partly reflect the interrelationships among age, professional experience, marital status, and position rather than independent associations.

The total intrapreneurship score above the scale midpoint indicates that nurses working in private hospitals have considerable potential to contribute to innovation, quality improvement, and organizational development. This finding is consistent with previous studies reporting moderate to high levels of entrepreneurial or intrapreneurial tendencies among nurses and healthcare professionals [33,35,38]. However, it should be interpreted within the conceptual distinction between entrepreneurship and intrapreneurship. While nurse entrepreneurship generally involves independent professional activity or venture creation, nurse intrapreneurship refers to entrepreneurial and innovation-oriented behaviors enacted within existing healthcare organizations, through which nurses can use initiative and innovation to improve services, processes, and organizational outcomes [11,13,28,29]. Therefore, the intrapreneurship level observed in this study may reflect nurses’ potential to initiate improvements, generate new ideas, and contribute to organizational change within the institutional context of private hospitals.

Among the subscales, innovativeness had the highest mean score. This finding indicates that nurses were open to new ideas and may be willing to contribute to practice improvement, research-oriented thinking, and technological or organizational innovation. Nurses are uniquely positioned to identify practical problems in care delivery because of their continuous contact with patients, families, and multidisciplinary teams. Previous research similarly emphasizes that nurses can be important sources of innovation in healthcare, particularly when organizational structures support and enable them to transform practice-based ideas into broader improvements [39,40]. A recent Türkiye-based study also reported high individual innovativeness among nurses and emphasized that education, support, and appropriate guidance are important for implementing innovations in nursing practice [41]. The high innovativeness score in this study is also consistent with studies reporting stronger tendencies toward innovativeness than toward other dimensions such as autonomy or risk-taking among nurses and other healthcare professionals [33,35,42].

Risk-taking and proactiveness also had a mean score above the scale midpoint. This finding suggests that nurses may be willing to take initiative, anticipate problems, and engage in change-oriented behavior, while still operating within the safety-sensitive boundaries of healthcare. In healthcare, risk-taking should not be interpreted as unsafe or uncontrolled action, but rather as a willingness to take calculated, responsible, and professionally grounded initiative. Previous literature suggests that considered risk-taking is closely related to innovation and that psychologically safe and managerially supportive work environments can encourage nurses to express new ideas, challenge ineffective routines, and participate in improvement efforts without fear of blame or failure [43,44,45,46].

In contrast, autonomy had the lowest mean score. This is one of the most important findings of the study because it reflects a gap between nurses’ willingness to innovate and their perceived capacity to act independently within organizational boundaries. Although nurses reported higher scores for innovativeness and risk-taking and proactiveness, their lower autonomy scores suggest that organizational and professional constraints may limit their ability to act independently, make decisions, or implement new ideas. This pattern is meaningful in hospital settings, where patient safety, standard procedures, hierarchical structures, and institutional roles necessarily shape professional action. However, excessive restriction of autonomy may prevent innovative ideas from being translated into practice. Previous studies emphasize that professional autonomy is central to nursing innovation and intrapreneurship because it enables nurses to use clinical judgment, challenge ineffective routines, and contribute to service improvement [47,48]. Other studies also highlight that organizational support, protected time, resources, and innovation-supportive climates influence whether nurses can move from having ideas to implementing them [13,39].

The pattern observed across the subscales suggests that nurses may possess strong innovative potential, but this potential may not always be fully supported by organizational conditions. In particular, the gap between high innovativeness and comparatively lower autonomy indicates that openness to innovation does not necessarily correspond to perceived freedom to act independently within organizational structures. Intrapreneurship depends not only on individual willingness, creativity, or proactivity, but also on organizational environments that provide autonomy, managerial support, resources, and recognition of innovative efforts. This interpretation is consistent with the broader intrapreneurship literature, which highlights the importance of supportive organizational climates, leadership, and opportunities for employee-driven innovation [14,15,16]. In private hospitals, where performance expectations, quality standards, and operational efficiency are particularly emphasized, strengthening nurses’ autonomy and participation in improvement processes may be essential for transforming intrapreneurial potential into concrete organizational outcomes.

Gender was the only characteristic that remained significantly associated with selected intrapreneurship outcomes after adjustment for other demographic and professional variables. However, the magnitude of these adjusted differences was small (partial η^2^ = 0.012–0.022; adjusted mean differences = 0.19–0.34 on the 1–5 scale), and male nurses represented only 17.0% of the sample. Therefore, these findings should not be interpreted as evidence of a substantial gender gap in intrapreneurship. Previous findings on gender differences in nurses’ entrepreneurship and intrapreneurship are inconsistent: some studies report no significant gender-based differences, whereas others identify limited differences in selected dimensions such as risk-taking, autonomy, or entrepreneurial tendency [33,34,35,38,49,50]. The present finding should therefore be interpreted cautiously. Because the study did not assess gender-related organizational or social mechanisms, the observed differences cannot be attributed to specific underlying causes. More broadly, corporate entrepreneurship literature indicates that entrepreneurial activity within organizations may be influenced by organizational context, autonomy, role expectations, institutional conditions, gender equality, cultural norms, and social expectations [15,51,52]. From a managerial perspective, equitable access to mentorship, innovation-related opportunities, leadership development, and organizational support for autonomous practice should be considered a general principle for supporting nurse intrapreneurship.

Marital status showed an unadjusted association with total intrapreneurship and autonomy, with single nurses scoring higher than married nurses. However, this association did not remain statistically significant after adjustment for other demographic and professional characteristics. Therefore, marital status should not be interpreted as an independent determinant of intrapreneurship in this sample. The unadjusted difference may partly reflect the overlap between marital status, age, professional experience, and role-related responsibilities. The literature provides mixed evidence on this issue. While some studies found no significant differences according to marital status, others reported higher risk-taking or autonomy among single participants, or higher innovativeness among married nurses [33,34,35,49,50,53]. These inconsistencies suggest that marital status may not have a direct or uniform relationship with intrapreneurship; rather, its influence may depend on work context, family responsibilities, organizational support, and the specific intrapreneurship dimension examined.

Age-related differences were observed only for autonomy in the unadjusted analyses. Age group was not significantly associated with total intrapreneurship, innovativeness, or risk-taking and proactiveness, whereas younger nurses had higher autonomy scores than nurses aged 40 years and above. However, age group was not independently associated with autonomy in the multivariable model. This suggests that the unadjusted age-related difference may partly reflect its overlap with professional experience, marital status, or professional position. Previous literature on age and entrepreneurial or innovative tendencies is also mixed. Some studies suggest that younger individuals may be more open to change, risk-taking, and independent action, whereas others indicate that older age may support innovation-related behaviors through professional experience, accumulated knowledge, and stronger workplace networks [23,35,38,49,54]. In the present study, age-related findings should therefore be interpreted as descriptive bivariate differences rather than independent effects.

Educational level appeared to be related mainly to innovativeness. In the unadjusted comparisons, postgraduate nurses had higher innovativeness scores than vocational health high school nursing graduates. This finding may be explained by the greater exposure of postgraduate nurses to research, evidence-based practice, critical thinking, and problem-solving approaches. However, educational level did not remain significantly associated with innovativeness after adjustment for other demographic and professional characteristics, and it was not independently associated with the total score or the other subscales. This suggests that advanced formal education may support innovative thinking, but may not be sufficient on its own to explain intrapreneurial tendencies when other workforce characteristics are considered. Previous literature indicates that entrepreneurship knowledge and skills are often insufficiently addressed in nursing education, while organizational culture, managerial support, practical opportunities, and innovation-related educational activities are also important for developing entrepreneurial, intrapreneurial, and innovative behavior [5,25,38,54]. Therefore, continuing education programs should not only promote innovation awareness but also include practical components related to initiative-taking, project development, leadership, organizational change, and the development of an intrapreneurial mindset. Recent nursing education literature similarly emphasizes that preparing nurses for increasingly complex healthcare roles requires the development of intrapreneurial skills, including leadership, cognitive flexibility, future orientation, and the ability to innovate within organizations [26].

No significant differences were found in intrapreneurship scores according to unit of employment. This finding suggests that intrapreneurial tendencies may not be limited to specific clinical settings such as emergency departments, inpatient units, intensive care units, outpatient units, or management units. Although previous studies on individual innovativeness have reported differences according to clinical settings or work-related characteristics [50,54], the absence of unit-based differences in the present study may reflect differences in the construct measured and the influence of common institutional policies, managerial structures, and organizational culture across units within private hospitals. Intrapreneurship in healthcare is shaped not only by individual characteristics but also by formal and informal institutional conditions, including autonomy, resources, managerial support, and opportunities for participation in decision-making [13,14,16]. From an organizational perspective, this finding is encouraging because intrapreneurship development programs can be designed for nurses across the entire institution rather than targeting only selected departments.

Professional position also showed unadjusted associations with total intrapreneurship, risk-taking and proactiveness, and autonomy. Post-hoc analyses showed that staff nurses had higher total intrapreneurship and autonomy scores than charge nurses. However, professional position did not remain significantly associated with any intrapreneurship outcome after adjustment. This indicates that the observed bivariate differences may partly reflect the interrelationships among position, age, professional experience, and organizational role expectations. Although nurse managers and leaders are often expected to support innovation, their formal leadership roles may require balancing change initiatives with operational priorities, staffing responsibilities, accountability for unit functioning, and institutional rules. The broader intrapreneurship literature also emphasizes that organizational position, formal structures, managerial support, and institutional constraints can influence whether employees feel able to engage in intrapreneurial action [13,25,29]. These findings suggest that leadership positions do not automatically translate into higher perceived autonomy for intrapreneurial action; rather, organizational structures should explicitly support both staff nurses and nurse leaders in innovation-related decision-making.

Professional experience was associated with autonomy only in the unadjusted comparisons. Nurses with 13–36 months of experience had higher autonomy scores than those with 121 months or more of experience. However, professional experience was not independently associated with autonomy or any other intrapreneurship outcome in the adjusted models. This pattern suggests that experience-related differences should be interpreted cautiously and may partly overlap with age, marital status, and professional position. The relationship between professional experience and entrepreneurship or innovation-related outcomes remains inconsistent in the literature. Some studies suggest that professional experience may be associated with entrepreneurial tendencies or individual innovativeness, whereas others report weaker or dimension-specific associations [23,31,35,38,54]. In the present study, professional experience appeared to be more relevant in the unadjusted autonomy comparisons than as an independent predictor of general intrapreneurial orientation.

The correlation analysis showed positive and statistically significant relationships between the total intrapreneurship score and all subscale scores. The strongest correlation was observed between the total scale and risk-taking and proactiveness, followed by autonomy and innovativeness. These findings are expected because the total score is calculated from the subscale items. However, the relatively weak correlation between innovativeness and autonomy is noteworthy. This pattern suggests that being open to new ideas does not necessarily mean that nurses feel able to act independently to implement them. In other words, innovativeness and autonomy may represent related but distinct aspects of intrapreneurship. This finding reinforces the main interpretation of the study: nurses may have strong innovative potential, but organizational conditions may limit their perceived autonomy to translate this potential into practice. Similar concerns have been raised in previous work showing that nurses may be willing to innovate but may lack the resources, protected time, and structural support needed to implement their ideas in practice [13,39].

Taken together, the adjusted findings suggest that nurses’ intrapreneurship should not be interpreted solely as an individual or demographic attribute. Although gender remained significantly associated with selected intrapreneurship outcomes, most bivariate differences were no longer statistically significant after adjustment, indicating that organizational conditions may be central to how nurses’ intrapreneurial potential is expressed. Recent studies support this interpretation by showing that head nurse empowerment, organizational climate, professional autonomy, managerial support, and cultural support are associated with nurses’ innovative behavior and innovation outputs [55,56,57]. These findings are consistent with the present study, in which autonomy was the lowest-scoring subscale and several demographic and professional differences were no longer statistically significant after adjustment.

### 4.2. Theoretical and Managerial Implications

The findings have both theoretical and managerial implications. From a theoretical perspective, the findings suggest that nurse intrapreneurship may be better interpreted within its organizational context rather than solely as an individual characteristic. Although gender remained significantly associated with selected outcomes after adjustment, most demographic and professional differences observed in the bivariate analyses were no longer statistically significant in the multivariable models. This suggests that future models of nurse intrapreneurship should more explicitly incorporate organizational conditions such as autonomy, managerial support, psychological safety, access to resources, participation in decision-making, and recognition of innovative efforts [55,56,57]. The limited replication of the original three-factor structure in the exploratory factor analysis also indicates that further conceptual and psychometric work is needed to examine how intrapreneurship is expressed among nurses and other healthcare professionals.

From a managerial perspective, the findings have several implications for nursing management in private hospitals. First, nurse managers should recognize that nurses’ intrapreneurial potential may not be fully captured by demographic or professional characteristics alone. Instead, organizational structures should be designed to support autonomy, participation in decision-making, and psychological safety for proposing and testing new ideas. Second, because autonomy was the lowest-scoring dimension, hospital managers should create structured mechanisms that allow nurses to transform innovative ideas into practice, such as mentorship programs, innovation committees, quality improvement projects, protected time for idea development, and recognition systems for innovations led by nurses. Recent Türkiye-based evidence among healthcare professionals also suggests that improving motivational and organizational factors, such as a suitable work environment, adequate equipment and resources, constructive managerial behavior, recognition, and opportunities for promotion and specialization, may support intrapreneurship [58]. More generally, equitable access to leadership opportunities, professional development, and innovation resources should remain a principle of nursing management, irrespective of the small gender-associated differences observed in this study. Finally, continuing education programs should include competencies that support intrapreneurship, such as taking initiative, calculated risk-taking, project development, leadership, organizational change, cognitive flexibility, future orientation, and innovation within organizations [26,55,56,57].

### 4.3. Study Limitations and Future Scope

This study has some limitations. Its cross-sectional design does not allow causal interpretation of the relationships between demographic and professional characteristics and intrapreneurship levels. In addition, the use of a convenience sampling method, online data collection, and the inclusion of nurses only from private hospitals may limit the generalizability of the findings to all nurses in Türkiye, particularly those working in public hospitals or other healthcare settings. The restriction to private-hospital respondents was made after data collection because public-sector participation was insufficient for meaningful analysis. Although recruitment was not restricted to a single healthcare group, respondents’ province of employment, hospital of employment, or healthcare-group identifiers were not collected; therefore, the exact geographic and institutional distribution of the final sample could not be determined. Furthermore, because the exact number of nurses who received the survey invitation could not be determined, the response rate could not be calculated. These issues should be considered when interpreting representativeness and potential selection bias. Moreover, all data were collected through self-report measures, which may be subject to social desirability or response bias. Another limitation concerns the measurement of intrapreneurship. Although the Intrapreneurship Scale showed good internal consistency in this sample, the exploratory factor analysis did not clearly reproduce the original three-dimensional structure of the scale among nurses. Therefore, the subscale findings should be interpreted cautiously, and further psychometric studies are needed to examine the dimensional structure of the scale in nursing and healthcare professional samples. Finally, although the study examined several demographic and professional characteristics, organizational variables such as leadership style, innovation climate, psychological safety, workload, staffing adequacy, managerial support, access to resources, recognition of innovative efforts, and participation in decision-making were not directly measured. For this reason, organizational explanations proposed in the discussion should be interpreted as hypotheses for future research rather than as direct findings of the present study. Future studies should use longitudinal, mixed-methods, and multi-center designs to examine how nurses’ intrapreneurial tendencies develop over time and how organizational factors and interventions may strengthen autonomy, innovation, and proactive behavior. Comparative studies involving public and private hospitals would also help clarify the structural and contextual factors that shape nurse intrapreneurship. In addition, two authors were affiliated with Acibadem Healthcare Group, and recruitment was conducted through professional nursing communication groups and e-mail lists; although recruitment was not restricted to a single healthcare group and no institutional identifiers were collected, this may have introduced selection bias and limits the ability to assess the institutional representativeness of the sample.

## 5. Conclusions

In conclusion, this study contributes to the limited empirical evidence on nurse intrapreneurship by focusing on employed nurses working within organizational settings rather than on entrepreneurship or entrepreneurial intention alone. The primary outcome, the total Intrapreneurship Scale score, was above the scale midpoint among nurses working in private hospitals in Türkiye. In secondary, exploratory analyses, innovativeness had the highest mean score, whereas autonomy had the lowest mean score and showed the most consistent differences in the bivariate analyses. Although several demographic and professional differences were observed in the bivariate analyses, most of these associations were no longer statistically significant after adjustment. This suggests that nurses’ intrapreneurial potential should not be interpreted only through individual or professional characteristics. Instead, perceived autonomy and organizational context should also be considered when seeking to understand how nurses’ innovative potential may be translated into intrapreneurial action. Strengthening nurses’ autonomy, participation in decision-making, managerial support, and innovation-friendly work environments may therefore help create conditions that support nurse intrapreneurship. In this sense, supporting nurse intrapreneurship is not only an individual competency issue, but also an organizational responsibility that can contribute to quality improvement, organizational development, and more adaptive healthcare systems. Future research should examine nurse intrapreneurship in different healthcare settings and use longitudinal or mixed-methods designs to better understand how individual, professional, and organizational factors interact to support or constrain intrapreneurial behavior.

## Figures and Tables

**Table 1:** Demographic and professional characteristics of nurses (*N* = 640).

Variable		*n* *	% *
Gender	Female	531	83.0
	Male	109	17.0
Age group	≤25	123	19.2
	26–30	251	39.2
	31–39	135	21.1
	≥40	131	20.5
Marital status	Single	318	49.7
	Married	322	50.3
Educational level **	Vocational health high school (nursing)	202	31.6
	Associate degree	163	25.5
	Bachelor’s degree	201	31.4
	Postgraduate degree	74	11.6
Unit of employment	Emergency department	80	12.5
	Outpatient unit	156	24.4
	Inpatient unit	220	34.4
	Intensive care unit	111	17.3
	Management	73	11.4
Position	Staff nurse	313	48.9
	Team leader	120	18.8
	Charge nurse	160	25.0
	Nurse manager	47	7.3
Professional experience	0–12 months	28	4.4
	13–36 months	68	10.6
	37–60 months	86	13.4
	61–120 months	159	24.8
	≥121 months	299	46.7

* *n* = frequency; % = percentage. ** Associate = 2-year college degree. Bachelor’s = 4-year university degree. Postgraduate = Master’s or Doctorate. Vocational health high school (nursing) refers to graduates of secondary-level nursing programs in Türkiye who are qualified to practice as nurses. This country-specific educational pathway should be considered when comparing educational categories across countries.

**Table 2:** Descriptive statistics for the Intrapreneurship Scale and its subscales.

Subscale	n	Mean ± SD	Median (IQR)	Min–Max
Innovativeness	640	4.27 ± 0.67	4.20 (0.80)	1.00–5.00
Risk-taking/Proactiveness	640	3.80 ± 0.69	3.80 (0.70)	1.00–5.00
Autonomy	640	3.04 ± 0.85	3.00 (1.29)	1.00–5.00
Total Scale	640	3.69 ± 0.61	3.67 (0.62)	1.00–5.00

Note: SD = standard deviation; IQR = interquartile range; Min–Max = minimum–maximum.

**Table 3:** Comparison of Intrapreneurship Scale and subscale scores according to demographic and professional characteristics.

Variable	Group	n	Total Scale Median (IQR)	Innovativeness Median (IQR)	Risk-Taking/Proactiveness Median (IQR)	Autonomy Median (IQR)
Gender	Female	531	3.67 (0.62)	4.20 (0.80)	3.80 (0.70)	3.00 (1.17)
	Male	109	3.90 (0.81)	4.20 (0.80)	4.00 (1.00)	3.33 (1.50)
					
	*p*		**<0.001**	0.456	**<0.001**	**0.001**
Age group	≤25	123	3.67 (0.67)	4.20 (0.80)	3.80 (0.90)	3.33 (1.17)
	26–30	251	3.71 (0.67)	4.40 (0.80)	3.80 (0.80)	3.00 (1.33)
	31–39	135	3.67 (0.62)	4.40 (0.80)	3.80 (0.70)	2.83 (1.17)
	≥40	131	3.62 (0.57)	4.20 (0.60)	3.80 (0.60)	2.67 (1.00)
					
	*p*		0.120	0.201	0.494	**<0.001**
Marital status	Single	318	3.71 (0.68)	4.20 (0.80)	3.80 (0.80)	3.17 (1.33)
	Married	322	3.67 (0.57)	4.40 (0.80)	3.80 (0.70)	2.83 (1.00)
					
	*p*		**0.044**	0.691	0.328	**<0.001**
Educational level	Vocational health high school (nursing)	202	3.67 (0.63)	4.20 (0.60)	3.90 (0.60)	3.00 (1.33)
	Associate	163	3.71 (0.62)	4.40 (0.80)	3.80 (0.70)	3.00 (1.50)
	Bachelor’s	201	3.67 (0.64)	4.20 (0.80)	3.70 (0.70)	3.00 (1.00)
	Postgraduate	74	3.71 (0.58)	4.40 (0.80)	3.80 (0.70)	2.67 (0.87)
					
	*p*		0.915	**0.024**	0.572	0.270
Unit of employment	Emergency	80	3.64 (0.65)	4.20 (0.60)	3.90 (0.90)	2.92 (1.50)
	Outpatient	156	3.71 (0.73)	4.20 (0.80)	3.80 (0.80)	3.00 (1.17)
	In-patient	220	3.67 (0.67)	4.20 (0.80)	3.80 (0.70)	3.00 (1.29)
	ICU	111	3.62 (0.62)	4.40 (0.80)	3.70 (0.50)	3.00 (1.17)
	Management	73	3.71 (0.57)	4.40 (0.60)	3.90 (0.65)	3.00 (0.83)
					
	*p*		0.704	0.286	0.261	0.931
Position	Staff nurse	313	3.71 (0.71)	4.20 (0.80)	3.90 (0.90)	3.17 (1.33)
	Team leader	120	3.62 (0.52)	4.20 (0.80)	3.70 (0.70)	3.00 (0.83)
	Charge nurse	160	3.60 (0.57)	4.40 (0.80)	3.80 (0.60)	2.75 (1.00)
	Nurse manager	47	3.76 (0.57)	4.40 (0.60)	4.00 (0.50)	2.83 (0.83)
					
	*p*		**0.025**	0.258	**0.017**	**0.001**
Professional experience	0–12 mo	28	3.76 (0.71)	4.20 (0.80)	3.80 (0.75)	3.25 (1.08)
	13–36 mo	68	3.81 (0.76)	4.20 (0.80)	4.00 (0.90)	3.33 (1.33)
	37–60 mo	86	3.69 (0.64)	4.20 (1.20)	3.80 (0.90)	3.00 (1.50)
	61–120 mo	159	3.67 (0.67)	4.20 (0.80)	3.80 (0.70)	3.00 (1.33)
	≥121 mo	299	3.62 (0.57)	4.40 (0.80)	3.80 (0.70)	2.83 (1.00)
					
	*p*		0.066	0.848	0.234	**0.001**

Note. Values are presented as median (IQR). IQR = interquartile range; ICU = intensive care unit; mo = months. Group comparisons were performed using the Mann–Whitney U test or Kruskal–Wallis test, as appropriate. Bold *p* values indicate statistical significance at *p* < 0.05. For Bonferroni-corrected post-hoc pairwise comparisons, *p* ≤ 0.008 was considered statistically significant for variables with four groups and *p* ≤ 0.005 for variables with five groups. Complete model results are provided in Supplementary Table S2, and the corresponding Mann–Whitney U and Kruskal–Wallis H statistics are reported in Supplementary Table S3.

**Table 4:** Significant predictors in the multivariable general linear models for the total Intrapreneurship Scale and subscale scores.

Dependent Variable	Significant Adjusted Predictor	F	*p*	Partial η^2^	B	95% CI for B
Total Intrapreneurship Scale	Gender: female vs. male	7.697	0.006	0.012	−0.186	−0.317 to −0.054
Innovativeness	None	—	—	—	—	—
Risk-taking & Proactiveness	Gender: female vs. male	8.759	0.003	0.014	−0.226	−0.375 to −0.076
Autonomy	Gender: female vs. male	14.235	<0.001	0.022	−0.344	−0.523 to −0.165

Note. Multivariable general linear models were adjusted for gender, age group, educational level, marital status, unit of employment, professional position, and professional experience. The reference category for gender was male. B values represent adjusted mean differences for female nurses compared with male nurses. Partial η^2^ = partial eta squared; CI = confidence interval. Complete model results for all fixed factors and category-specific parameter estimates are provided in Supplementary Table S2.

**Table 5:** Spearman correlations between the Intrapreneurship Scale and its subscales (N = 640).

Variable	1	2	3	4
1. Total Intrapreneurship Scale	1.000	-	-	-
2. Innovativeness	0.585 **	1.000	-	-
3. Risk-taking & Proactiveness	0.906 **	0.564 **	1.000	-
4. Autonomy	0.749 **	0.139 **	0.484 **	1.000

Note. Spearman’s rho correlation analysis was used. ** *p* < 0.01.

## Data Availability

The data presented in this study are available on request from the corresponding author due to privacy and ethical restrictions.

## Supplementary Materials

The following supporting information can be downloaded at: https://www.mdpi.com/article/10.3390/healthcare14213684/s1, Table S1: Exploratory factor analysis of the 21-item Intrapreneurship Scale; Table S2: Complete multivariable general linear models; Table S3: Mann–Whitney U and Kruskal–Wallis H test statistics corresponding to manuscript Table 3.

## Appendix A. Study Variables and Questionnaire Items

**Table A1:** Demographic and professional variables used in the study.

Variable	Categories/Measurement
Year of birth/age	Year of birth was collected and categorized into age groups for analysis: ≤25, 26–30, 31–39, and ≥40 years
Gender	Female; Male
Educational level	Vocational health high school (nursing); Associate degree; Bachelor’s degree; Postgraduate degree
Marital status	Single; Married
Unit of employment	Emergency department; Outpatient unit; Inpatient unit; Intensive care unit; Management
Professional position	Staff nurse; Team leader; Charge nurse; Nurse manager
Professional experience	0–12 months; 13–36 months; 37–60 months; 61–120 months; ≥121 months
Type of institution	Private hospital

**Table A2:** Intrapreneurship Scale items used in the study.

Item no.	Item
1	I think I am someone who is open to new ideas.
2	I motivate my colleagues to be innovative.
3	I strive to do new things related to my work.
4	I believe that delegating authority to subordinates helps foster creative ideas.
5	I attach importance to research and development activities, technological leadership, and innovation.
6	I believe that a situation of uncertainty presents an opportunity to take initiative.
7	I am successful in using organizational resources, such as time, money, and human resources, to make use of opportunities in my work environment.
8	I do not hesitate to make major changes to a plan I have worked hard on, if necessary.
9	If I believe in the success of a task, I can take on any risk.
10	I have a strong tendency toward high-risk projects.
11	When making decisions in situations involving uncertainty, I adopt a bold attitude to maximize potential high returns.
12	Instead of monitoring developments to evaluate activities in my work environment, I try to stay ahead of them.
13	I do not let developments direct me; instead, I try to direct them.
14	I would like to participate in long-term planning activities at my workplace with strong imagination and determination.
15	I strive to be at the forefront of developing new practices, products, techniques, or technologies at my workplace.
16	In professional life, I follow my own path without worrying about what others think.
17	I prefer working alone on any task.
18	When working on a new project, I have complete freedom to choose the direction I will take.
19	When I put forward a new idea, I do not wait for others’ approval.
20	When developing an innovation related to my work, I can make decisions without consulting my superiors.
21	When developing an innovation related to my work, I may deviate from standard operating procedures and organizational rules.

Response options: 1 = Strongly disagree; 2 = Disagree; 3 = Neutral; 4 = Agree; 5 = Strongly agree.
