# Supplementary material for: Intrapreneurship Among Nurses in Private Hospitals According to Demographic and Professional Characteristics: A Cross-Sectional Study in Türkiye
Source: Healthcare (Basel). 2026 Oct 30;14(21):3684. doi: 10.3390/healthcare14213684 (PMC13763365; doi:10.3390/healthcare14213684)
Supplement: Supplementary file 1 [file healthcare-14-03684-s001.zip › healthcare-4529049-supplementary.pdf]

Supplementary Tables

Intrapreneurship Among Nurses in Private Hospitals According to Demographic and Professional Characteristics: A Cross-Sectional Study in Türkiye

Manuscript ID: healthcare-4529049

Table S1. Exploratory factor analysis of the 21-item Intrapreneurship Scale

Item*	Factor 1	Factor 2	Factor 3	Initial h²	Extracted h²
1	0.768	—	—	0.626	0.662
2	0.731	—	0.310	0.601	0.631
3	0.774	—	0.324	0.659	0.699
4	0.557	—	0.365	0.475	0.473
5	0.749	—	0.357	0.645	0.691
6	0.589	—	—	0.388	0.369
7	0.737	—	—	0.507	0.509
8	0.730	—	—	0.556	0.525
9	0.705	—	—	0.557	0.496
10	0.689	—	—	0.553	0.501
11	0.714	—	—	0.554	0.564
12	0.447	—	—	0.496	0.438
13	0.483	—	—	0.528	0.441
14	0.724	—	—	0.596	0.521
15	0.715	—	—	0.603	0.542
16	0.442	—	—	0.437	0.411
17	—	0.416	—	0.302	0.279
18	0.477	—	—	0.445	0.451
19	—	0.685	—	0.478	0.525
20	—	0.946	—	0.634	0.793
21	—	0.805	—	0.548	0.569

*Principal axis factoring with oblimin rotation and Kaiser normalization; three factors specified. Loadings with |value| < 0.30 were suppressed in the SPSS output and are shown as dashes. *Item wording appears in manuscript Appendix A, Table A2. N = 640; KMO = 0.929; Bartlett's $\chi^2(210) = 7388.364$, $p < 0.001$.*

Factor	Initial eigenvalue	Initial variance %	Initial cumulative %	Extracted SS loading	Extracted variance %	Extracted cumulative %	Rotated SS loading
1	8.540	40.669	40.669	8.077	38.460	38.460	7.915
2	2.812	13.389	54.058	2.402	11.438	49.898	3.571
3	1.038	4.941	58.999	0.612	2.915	52.813	1.185

The three extracted factors explain 52.813% of variance. For the oblique rotation, rotated sums of squared loadings cannot be summed to obtain a percentage of total variance.

Table S2. Complete multivariable general linear models

Table S2a. Type III tests of fixed factors

Total Intrapreneurship Scale

Effect	df	F	p	Partial η²
Full model	19	1.298	0.177	0.038
Intercept	1	4977.462	<0.001	0.889
Gender	1	7.697	0.006	0.012
Age group	3	1.406	0.240	0.007
Educational level	3	0.822	0.482	0.004
Marital status	1	1.668	0.197	0.003
Unit of employment	4	0.196	0.940	0.001
Professional position	3	0.405	0.749	0.002
Professional experience	4	0.278	0.892	0.002

$R^2 = 0.038$; adjusted $R^2 = 0.009$. Error df = 620; N = 640.

Innovativeness

Effect	df	F	p	Partial η²
Full model	19	0.777	0.736	0.023
Intercept	1	5162.710	<0.001	0.893
Gender	1	1.274	0.260	0.002
Age group	3	1.115	0.342	0.005
Educational level	3	0.954	0.414	0.005
Marital status	1	0.631	0.427	0.001
Unit of employment	4	0.097	0.983	0.001
Professional position	3	0.085	0.968	0.000
Professional experience	4	0.629	0.642	0.004

$R^2 = 0.023$; adjusted $R^2 = -0.007$. Error df = 620; N = 640.

Risk-taking and Proactiveness

Effect	df	F	p	Partial η²
Full model	19	1.301	0.175	0.038
Intercept	1	4148.360	<0.001	0.870
Gender	1	8.759	0.003	0.014
Age group	3	1.299	0.274	0.006
Educational level	3	0.803	0.493	0.004
Marital status	1	0.369	0.544	0.001
Unit of employment	4	0.298	0.880	0.002
Professional position	3	0.748	0.524	0.004
Professional experience	4	0.354	0.841	0.002

$R^2 = 0.038$; adjusted $R^2 = 0.009$. Error df = 620; N = 640.

Autonomy

Effect	df	F	p	Partial η²
Full model	19	2.682	<0.001	0.076
Intercept	1	1840.951	<0.001	0.748
Gender	1	14.235	<0.001	0.022
Age group	3	1.639	0.179	0.008
Educational level	3	0.668	0.572	0.003
Marital status	1	3.745	0.053	0.006
Unit of employment	4	0.354	0.841	0.002
Professional position	3	0.778	0.507	0.004
Professional experience	4	0.921	0.451	0.006

$R^2 = 0.076$; adjusted $R^2 = 0.048$. Error df = 620; N = 640.

Table S2b. Adjusted parameter estimates by category

Total Intrapreneurship Scale

Parameter (reference is final category)	B	95% CI for B	p
Intercept	3.894	3.659 to 4.130	<0.001
Gender: Female	−0.186	−0.317 to −0.054	0.006
Gender: Male	0 (reference)	—	—
Age group: ≤25 y	0.127	−0.081 to 0.335	0.230
Age group: 26–30 y	0.162	−0.007 to 0.331	0.060
Age group: 31–39 y	0.131	−0.022 to 0.283	0.093
Age group: ≥40 y	0 (reference)	—	—
Educational level: Vocational health high school (nursing)	−0.116	−0.300 to 0.069	0.218
Educational level: Associate	−0.132	−0.324 to 0.061	0.179
Educational level: Bachelor’s	−0.056	−0.232 to 0.120	0.532
Educational level: Postgraduate	0 (reference)	—	—
Marital status: Single	0.071	−0.037 to 0.179	0.197
Marital status: Married	0 (reference)	—	—
Unit of employment: Emergency	−0.089	−0.349 to 0.171	0.502
Unit of employment: Outpatient	−0.075	−0.312 to 0.161	0.532
Unit of employment: Inpatient	−0.097	−0.328 to 0.134	0.410
Unit of employment: Intensive care	−0.097	−0.332 to 0.139	0.420
Unit of employment: Management	0 (reference)	—	—
Professional position: Staff nurse	−0.017	−0.287 to 0.253	0.901
Professional position: Team leader	−0.076	−0.323 to 0.171	0.546
Professional position: Charge nurse	−0.075	−0.348 to 0.199	0.593
Professional position: Nurse manager	0 (reference)	—	—
Professional experience: 0–12 mo	0.068	−0.227 to 0.364	0.649
Professional experience: 13–36 mo	0.088	−0.119 to 0.295	0.405
Professional experience: 37–60 mo	0.001	−0.180 to 0.182	0.991
Professional experience: 61–120 mo	−0.007	−0.154 to 0.140	0.926
Professional experience: ≥121 mo	0 (reference)	—	—

Innovativeness

Parameter (reference is final category)	B	95% CI for B	p
Intercept	4.341	4.080 to 4.602	<0.001
Gender: Female	0.084	−0.062 to 0.229	0.260
Gender: Male	0 (reference)	—	—
Age group: ≤25 y	−0.030	−0.260 to 0.200	0.796
Age group: 26–30 y	0.046	−0.142 to 0.233	0.632
Age group: 31–39 y	0.124	−0.045 to 0.293	0.149
Age group: ≥40 y	0 (reference)	—	—
Educational level: Vocational health high school (nursing)	−0.165	−0.369 to 0.038	0.111
Educational level: Associate	−0.130	−0.343 to 0.082	0.229
Educational level: Bachelor's	−0.083	−0.278 to 0.112	0.406
Educational level: Postgraduate	0 (reference)	—	—
Marital status: Single	0.048	−0.071 to 0.168	0.427
Marital status: Married	0 (reference)	—	—
Unit of employment: Emergency	−0.079	−0.367 to 0.208	0.589
Unit of employment: Outpatient	−0.048	−0.310 to 0.215	0.721
Unit of employment: Inpatient	−0.034	−0.290 to 0.223	0.797
Unit of employment: Intensive care	−0.031	−0.292 to 0.230	0.816
Unit of employment: Management	0 (reference)	—	—
Professional position: Staff nurse	−0.058	−0.357 to 0.242	0.705
Professional position: Team leader	−0.070	−0.343 to 0.204	0.618
Professional position: Charge nurse	−0.059	−0.362 to 0.244	0.701
Professional position: Nurse manager	0 (reference)	—	—
Professional experience: 0–12 mo	0.089	−0.238 to 0.417	0.592
Professional experience: 13–36 mo	−0.073	−0.302 to 0.156	0.532
Professional experience: 37–60 mo	−0.046	−0.246 to 0.154	0.653
Professional experience: 61–120 mo	0.053	−0.110 to 0.216	0.523
Professional experience: ≥121 mo	0 (reference)	—	—

Risk-taking and Proactiveness

**Parameter (reference is final category)**	**B**	**95% CI for B**	**p**
Intercept	4.145	3.876 to 4.413	<0.001
Gender: Female	−0.226	−0.375 to −0.076	0.003
Gender: Male	0 (reference)	—	—
Age group: ≤25 y	0.103	−0.133 to 0.340	0.391
Age group: 26–30 y	0.177	−0.016 to 0.369	0.072
Age group: 31–39 y	0.121	−0.053 to 0.294	0.172
Age group: ≥40 y	0 (reference)	—	—
Educational level: Vocational health high school (nursing)	−0.129	−0.338 to 0.081	0.228
Educational level: Associate	−0.172	−0.391 to 0.047	0.123
Educational level: Bachelor’s	−0.109	−0.309 to 0.091	0.286
Educational level: Postgraduate	0 (reference)	—	—
Marital status: Single	0.038	−0.085 to 0.161	0.544
Marital status: Married	0 (reference)	—	—
Unit of employment: Emergency	−0.023	−0.318 to 0.273	0.880
Unit of employment: Outpatient	−0.050	−0.320 to 0.220	0.716
Unit of employment: Inpatient	−0.098	−0.361 to 0.166	0.467
Unit of employment: Intensive care	−0.090	−0.358 to 0.178	0.510
Unit of employment: Management	0 (reference)	—	—
Professional position: Staff nurse	−0.117	−0.425 to 0.190	0.455
Professional position: Team leader	−0.188	−0.470 to 0.093	0.189
Professional position: Charge nurse	−0.160	−0.471 to 0.151	0.313
Professional position: Nurse manager	0 (reference)	—	—
Professional experience: 0–12 mo	0.123	−0.214 to 0.459	0.475
Professional experience: 13–36 mo	0.114	−0.121 to 0.350	0.341
Professional experience: 37–60 mo	0.017	−0.188 to 0.223	0.868
Professional experience: 61–120 mo	−0.002	−0.170 to 0.165	0.979
Professional experience: ≥121 mo	0 (reference)	—	—

Autonomy

**Parameter (reference is final category)**	**B**	**95% CI for B**	**p**
Intercept	3.106	2.785 to 3.426	<0.001
Gender: Female	−0.344	−0.523 to −0.165	<0.001
Gender: Male	0 (reference)	—	—
Age group: ≤25 y	0.298	0.015 to 0.581	0.039
Age group: 26–30 y	0.235	0.005 to 0.465	0.046
Age group: 31–39 y	0.152	−0.055 to 0.360	0.150
Age group: ≥40 y	0 (reference)	—	—
Educational level: Vocational health high school (nursing)	−0.052	−0.303 to 0.198	0.683
Educational level: Associate	−0.065	−0.327 to 0.197	0.626
Educational level: Bachelor’s	0.054	−0.186 to 0.294	0.658
Educational level: Postgraduate	0 (reference)	—	—
Marital status: Single	0.145	−0.002 to 0.293	0.053
Marital status: Married	0 (reference)	—	—
Unit of employment: Emergency	−0.207	−0.561 to 0.147	0.251
Unit of employment: Outpatient	−0.141	−0.464 to 0.182	0.391
Unit of employment: Inpatient	−0.149	−0.464 to 0.166	0.353
Unit of employment: Intensive care	−0.163	−0.483 to 0.158	0.319
Unit of employment: Management	0 (reference)	—	—
Professional position: Staff nurse	0.183	−0.185 to 0.551	0.329
Professional position: Team leader	0.106	−0.230 to 0.443	0.536
Professional position: Charge nurse	0.055	−0.317 to 0.427	0.772
Professional position: Nurse manager	0 (reference)	—	—
Professional experience: 0–12 mo	−0.039	−0.442 to 0.363	0.849
Professional experience: 13–36 mo	0.178	−0.104 to 0.460	0.216
Professional experience: 37–60 mo	0.013	−0.234 to 0.259	0.919
Professional experience: 61–120 mo	−0.065	−0.265 to 0.135	0.525
Professional experience: ≥121 mo	0 (reference)	—	—

All seven fixed factors were entered simultaneously. Each B is the adjusted difference from the final listed category of the same factor; rows marked "0 (reference)" are fixed to zero and have no inferential statistics. Intercept is retained for completeness. p values are from SPSS and values reported as 0.000 are displayed as <0.001. CI = confidence interval; df = degrees of freedom; η^2 = eta squared. The total scale is the primary outcome; subscale models are secondary, exploratory analyses.

Table S3. Mann–Whitney U and Kruskal–Wallis H test statistics corresponding to manuscript Table 3.

Grouping variable	Test	Total	Innovativeness	Risk-taking / Proactiveness	Autonomy
Gender	U	22551.000	27645.500	21109.500	23007.000
Age group	H	5.835	4.635	2.397	18.837
Marital status	U	46493.000	50280.000	48913.000	42974.500
Educational level	H	0.516	9.414	2.001	3.925
Unit of employment	H	2.171	5.012	5.262	0.851
Position	H	9.380	4.034	10.250	15.645
Professional experience	H	8.826	1.377	5.570	19.054

Mann–Whitney U tests were used for binary grouping variables; Kruskal–Wallis H tests were used for variables with more than two groups. Corresponding p values and group medians (IQR) are reported in manuscript Table 3.
